# A Comprehensive Reactive-Oxygen-Species-Scavenging Metal–Organic Framework Orchestrates the AMPK–DOT1L–H3K79me3 Cascade to Alleviate Osteoarthritis

**DOI:** 10.34133/bmr.0402

**Published:** 2026-08-11

**Authors:** Yu Jin, Cheng Zhu, Tiancheng Li, Ruomei Li, Chengxiao Liu, Yixin Li, Bing Fang, Lunguo Xia

**Affiliations:** Department of Orthodontics, Shanghai Ninth People’s Hospital, Shanghai Jiao Tong University School of Medicine; College of Stomatology, Shanghai Jiao Tong University; National Center for Stomatology; National Clinical Research Center for Oral Diseases, Shanghai Key Laboratory of Stomatology, Shanghai 200011, China.

## Abstract

Osteoarthritis (OA) is a complex pathological condition characterized by oxidative stress and progressive cartilage breakdown. The reciprocal relationship between the inflammatory joint milieu and impaired chondrocyte function drives the progressive deterioration of OA. Inspired by natural metalloenzymes that utilize metal ions as catalytic centers, stable metal–organic frameworks (MOFs) assembled from natural polyphenols and metal ions have emerged as promising candidates for mitigating inflammatory diseases. Nonetheless, numerous nanozymes are limited to restricted antioxidant capacity, failing to eliminate various kinds of reactive oxygen species (ROS). To address these limitations, we engineered the MnO_2_@UiO-66(Ce) (abbreviated as MCU) system, which was fabricated through the integration of MnO_2_ into nanoscale mesoporous UiO-66 MOFs for OA therapy. Within the MOF architecture, MnO_2_ coupled with Ce clusters establishes a continuous superoxide dismutase/catalase cascade reaction platform that enables efficient ROS scavenging. In vitro and in vivo experiments reveal that the MCU system significantly reduces intracellular ROS accumulation and ameliorates the inflammatory microenvironment, consequently attenuating cartilage matrix destruction. Further mechanistic investigations indicates that MCU alleviates OA progression through the epigenetic activation of Wnt/β-catenin via adenosine monophosphate-activated protein kinase–disruptor of telomeric silencing 1-like-mediated H3K79 methylation. In summary, this study suggests that this highly efficient cascade catalytic system may represent a promising strategic avenue for combating oxidative stress in chronic inflammatory diseases.

## Introduction

Osteoarthritis (OA) ranks among the most prevalent degenerative joint disorders, imposing a profound and growing clinical and socioeconomic burden worldwide [1]. Since articular cartilage lacks intrinsic regenerative capacity, structural deterioration of OA is generally considered irreversible. As the disease advances, progressive cartilage loss culminates in impaired joint function and ultimately leads to joint dysfunction or disability [2]. At present, clinical strategies for OA focus on attenuating inflammation and alleviating pain, which cannot effectively and completely prevent disease progression [3].

In recent years, increasing studies have implicated that increased levels of various reactive oxygen species (ROS), including •O_2_^−^, •OH, and H_2_O_2_, contribute to an oxidative joint milieu that drives pathological changes [4,5]. Local oxidative stress within the articular cartilage microenvironment leads to degradation of the extracellular cartilage matrix. Local oxidative stress in the inflammatory joint milieu culminates in catabolism of the cartilage extracellular matrix and synovial inflammation, thereby accelerating disease progression [6]. The interplay between ROS and cartilage degeneration creates a vicious cycle, impeding cartilage repair and worsening OA symptoms. Hence, the key to OA therapy hinges on neutralizing multiple types of ROS and attenuating the proinflammatory microenvironment. Therapeutic approaches should therefore combine broad-spectrum ROS scavengers with anti-inflammatory interventions to restore joint homeostasis and promote effective long-term cartilage repair.

Endogenous antioxidant enzymes, including superoxide dismutase (SOD), constitute a vital component of the human antioxidant defense system [7]. While the therapeutic potential of exogenous SOD has been demonstrated, its clinical application is hindered by its intrinsic instability and rapid clearance from the human body. Catalytic biomaterials, biocompatible substances engineered to mimic enzyme activities that exhibit enhanced stability and tunability, have opened new frontiers for therapeutic interventions [8–10]. Recent advances have led to the development of single-atom nanozymes featuring metal–nitrogen coordination motifs to replicate the catalytic functions of natural enzymes [11,12]. Despite exhibiting potent catalase (CAT)-mimetic ability that supports antioxidant defenses and oxygen generation, these nanozymes fail to scavenge highly reactive •OH within the OA inflammatory milieu [13]. Consequently, engineering nanozymes with potent broad-spectrum antioxidant functions to eliminate multiple reactive oxygen species simultaneously is essential for effective OA treatment [14]. Furthermore, the regulatory mechanism through which nanozymes mitigate OA remains to be elucidated.

Metal–organic frameworks (MOFs) with porous periodic coordination polymers linked by metal ions and organic linkers have gained prominence as nanozymes, particularly owing to their structural tunability, biodegradability, and catalytic functionality for ROS elimination [15,16]. In recent years, growing evidence has highlighted the therapeutic promise of MOFs in treating inflammatory diseases [17,18]. A widely adopted strategy to improve the efficiency of ROS-scavenging processes involves the design of cascade reaction networks. This biomimetic approach mirrors the native antioxidant cascade that begins with the SOD-catalyzed dismutation of •O_2_^−^ into H_2_O_2_, which subsequently undergoes decomposition into water and molecular oxygen via CAT catalysis [19]. Among diverse MOFs, UiO-66(Ce)-type MOFs exhibit distinct structural advantages, including accessible Ce–O clusters and interconnected mesochannels that facilitate mass transport.

The multivalent nature of cerium ions confers nanozymes with robust SOD-mimetic behavior, while Ce-based clusters serve as active sites for rapid H_2_O_2_ binding [20]. Moreover, the excellent tunability of Ce allows for the integration of complementary catalytic motifs. For instance, CAT-mimicking components such as manganese dioxide (MnO_2_) can be incorporated, enabling the design of integrated SOD/CAT cascade nanozymes [21].

In the present study, we engineered an efficient scavenging system termed MnO_2_@UiO-66(Ce) (abbreviated as MCU) through the introduction of MnO_2_ into nanoscale mesoporous UiO-66 for OA therapy. The MCU system possesses outstanding multifaceted antioxidant defense properties. First, the UiO-66 system displays SOD-like capacity while simultaneously capturing H_2_O_2_; second, MnO_2_ acts as a CAT mimic, allowing spatially coupled catalytic decomposition of ROS. Our experimental results demonstrated that MCU acts as an effective ROS scavenger, modifying the articular cavity microenvironment to reduce extracellular matrix degradation and inflammatory responses, thereby slowing OA progression. Additionally, the underlying epigenetic mechanism through which MCU modulates OA disease was elucidated.

## Materials and Methods

### Preparation and property characterization of MCU

MCU was prepared as previously reported [22]. UiO-66 was first prepared by dissolving F127 (112.5 mg) in deionized water (27 mL), followed by the sequential addition of acetic acid (0.3375 mL), NaClO_4_·H_2_O (2,250 mg), and HNO_3_ (0.675 mL) under continuous stirring. (NH^4^)_2_Ce(NO_3_)_6_ (616.5 mg) and 1,4-dicarboxybenzene (186.75 mg) were subsequently added and stirred at 60 °C. The obtained F127@UiO-66 intermediate was washed and suspended in KMnO_4_ solution (0.1 mg/mL, 30 mL) with continuous stirring for 1 h. To remove F127, the product was subjected to a sequential purification process involving washes with *N*,*N*-dimethylformamide followed by immersion in ethanol at 60 °C for 48 h, with the ethanol being replaced daily.

Transmission electron microscopy (TEM) and energy-dispersive spectroscopy mapping were performed with a Talos F200× G2 microscope (FEI, USA). The hydrodynamic particle size was determined by dynamic light scattering (Zetasizer Nano ZS90; Malvern, UK). X-ray photoelectron spectroscopy (XPS) spectra was performed using a Nexsa XPS System (Thermo Fisher, USA), and x-ray diffraction analysis was completed by Empyrean (Panalytical, Netherlands). The SOD-like capacity was quantified with a total SOD activity detection kit (Beyotime, China). Dissolved O_2_ concentrations were monitored with an oxygen electrode in 10 mM H_2_O_2_ solutions, and the H_2_O_2_ elimination capacity was determined via an Amplex Red assay (Beyotime, China).

### Primary chondrocyte isolation and culture

Primary chondrocytes were obtained from the articular cartilage of 3-week-old Sprague–Dawley (SD) rats. Cartilage fragments were first treated with trypsin–EDTA, followed by further dissociation using 0.3% type II collagenase. The harvested cells were cultured in Dulbecco’s Modified Eagle Medium/F12 medium, and all subsequent assays were conducted using first- to third-passage chondrocytes. To mimic osteoarthritic conditions in vitro, primary chondrocytes were incubated with interleukin-1 beta (IL-1β; PeproTech, USA).

### Cytocompatibility evaluation of MCU

MCU biocompatibility was assessed through a Cell Counting Kit-8 (CCK-8; New Cell & Molecular Biotech, China). Primary chondrocytes were exposed to varying concentrations of MCU, and 24, 48, and 72 h later, the chondrocytes were rinsed with phosphate-buffered saline and treated with CCK-8 solution for 2 h at 37 °C. The absorbance at 450 nm was measured with an ELX Ultra microplate reader (BioTek, USA). A live/dead assay was performed with a calcein-AM/PI staining kit (Beyotime, China), and the cells were imaged with an inverted fluorescence microscope (ZEISS, Germany).

### Assessment of intracellular ROS levels in vitro

Intracellular ROS levels were quantified using a DCFH-DA assay (Beyotime, China), while mitochondrial superoxide levels were measured by a MitoSOX Red assay (YEASEN, China). Fluorescence signals were visualized and recorded with an inverted fluorescence microscope (ZEISS, Germany).

### Toluidine blue and safranin O staining

Primary chondrocytes were fixed in 4% paraformaldehyde to preserve the cellular architecture. Following fixation, the samples were rinsed with phosphate-buffered saline to remove residual fixative. To contrast matrix and cellular features, the specimens were stained either with 0.1% toluidine blue for 10 min to reveal sulfated proteoglycans or with 1% safranin O for 10 min to highlight cartilage matrix constituents. The samples were air-dried at room temperature and examined under an inverted microscope for detailed assessment of chondrocyte morphology and extracellular matrix organization.

### RNA isolation and qRT-PCR

Total RNA was extracted from cells using a commercial RNA extraction kit (Fastagen, China) according to the manufacturer’s protocol. Subsequently, cDNA was synthesized from the purified RNA with a cDNA synthesis kit (YEASEN, China). qRT-PCR was performed on a LightCycler instrument (Roche, Switzerland) with SYBR Green Master Mix (YEASEN, China). Quantitative analysis of the experimental results was conducted using the ΔΔCt method, with β-actin serving as the internal control. All primer sequences utilized in this study are provided in Table S1.

### Immunofluorescence staining

Following fixation in 4% paraformaldehyde and permeabilization with 0.1% Triton X-100, primary chondrocytes were immunostained with primary antibodies against NRF2 (Proteintech, USA), HO-1 (Proteintech, USA), ACAN (Abcam, USA), and SOX9 (Abcam, USA) overnight at 4 °C. The samples were then incubated with Alexa Fluor 647-conjugated secondary antibodies for 1 h in the dark. Nuclei were visualized with DAPI (YEASEN, China). Fluorescence images were acquired on a fluorescence microscope (ZEISS, Germany).

### Immunoblotting

Standard immunoblotting procedures were employed as previously reported [23]. The membranes were probed overnight at 4 °C with antibodies to NRF2 (Proteintech, USA), HO-1 (Proteintech, USA), p-AMPKα (CST, UK), AMPKα (CST, UK), DOT1L (CST, UK), H3K79me3 (CST, UK), β-catenin (Proteintech, USA), histone H3 (CST, UK), and β-actin (Proteintech, USA). The protein bands were visualized with horseradish-peroxidase-conjugated secondary antibodies on an Amersham 600 chemiluminescence system, and the band intensities were quantified with ImageJ software.

### RNA-sequencing analysis

RNA-sequencing (RNA-seq) libraries were sequenced on an Illumina NovaSeq 6000. DESeq2 was employed to normalize the data and calculate fold changes and *P* values. Kyoto Encyclopedia of Genes and Genomes pathway enrichment analysis was completed by the clusterProfiler R package (v3.18.1) according to differentially expressed genes (DEGs).

### OA animal model

The animal experiments were approved by the Ethics Board of Shanghai Ninth People’s Hospital affiliated to Shanghai Jiao Tong University School of Medicine. An animal model of OA was established by employing the anterior cruciate ligament transection (ACLT) procedure [24]. SD rats were divided into 3 groups (Sham, ACLT, and ACLT + MCU), and intra-articular injections started at the second week since ACLT surgery (twice 1 wk). The rats were sacrificed at the eighth week, after which the tissues were collected for further assays.

### Histological analysis and immunohistochemistry assay

Following fixation in 4% paraformaldehyde and decalcification in 10% EDTA, tissue samples were embedded in paraffin and sectioned. Histological evaluations were carried out using hematoxylin and eosin, safranin O–fast green, and toluidine blue staining assay. The results were assessed according to the Osteoarthritis Research Society International grading criteria. Immunohistochemical analysis followed a standard protocol involving deparaffinization, dehydration, peroxidase quenching with 0.3% hydrogen peroxide, and proteinase-K-mediated antigen retrieval. The sections were then incubated overnight at 4 °C with antibodies of ACAN (Abcam USA), SOX9 (Abcam USA), DOT1L (CST, UK), and β-catenin (Proteintech USA). After secondary antibody incubation, immunoreactivity was visualized with DAB substrate, followed by counterstaining with hematoxylin. Images were acquired via light microscope (Nikon, Japan) and quantitatively analyzed with ImageJ.

### Statistical analysis

Statistical analyses were conducted with GraphPad Prism 8.0. Intergroup comparisons were performed by Student *t* test (2 groups) or one-way analysis of variance (multiple groups), with a significance threshold of *P* <0.05.

## Results and Discussion

### Preparation and property characterization of MCU

During MCU synthesis, F127 functioned as a structural template to direct the assembly of UiO-66 with organized mesoporous channels. To achieve nanoscale particle dimensions, preserve excellent dispersion, and retain crystalline framework integrity, we precisely optimized the precursor concentration, controlled the introduction of HNO_3_, and systematically adjusted the Ce/F127 ratio. To integrate metal oxide components with enhanced catalytic performance, we employed an in situ reduction-deposition strategy that utilized F127 micelles confined within UiO-66 mesochannels as reducing agents for KMnO_4_. The Ce–O defect sites within the framework served as anchoring sites that effectively captured the reduced manganese species, promoting the formation of homogeneously distributed MnO_2_ nanoparticles with increased accessibility throughout the mesoporous network.

**Fig. 1.**
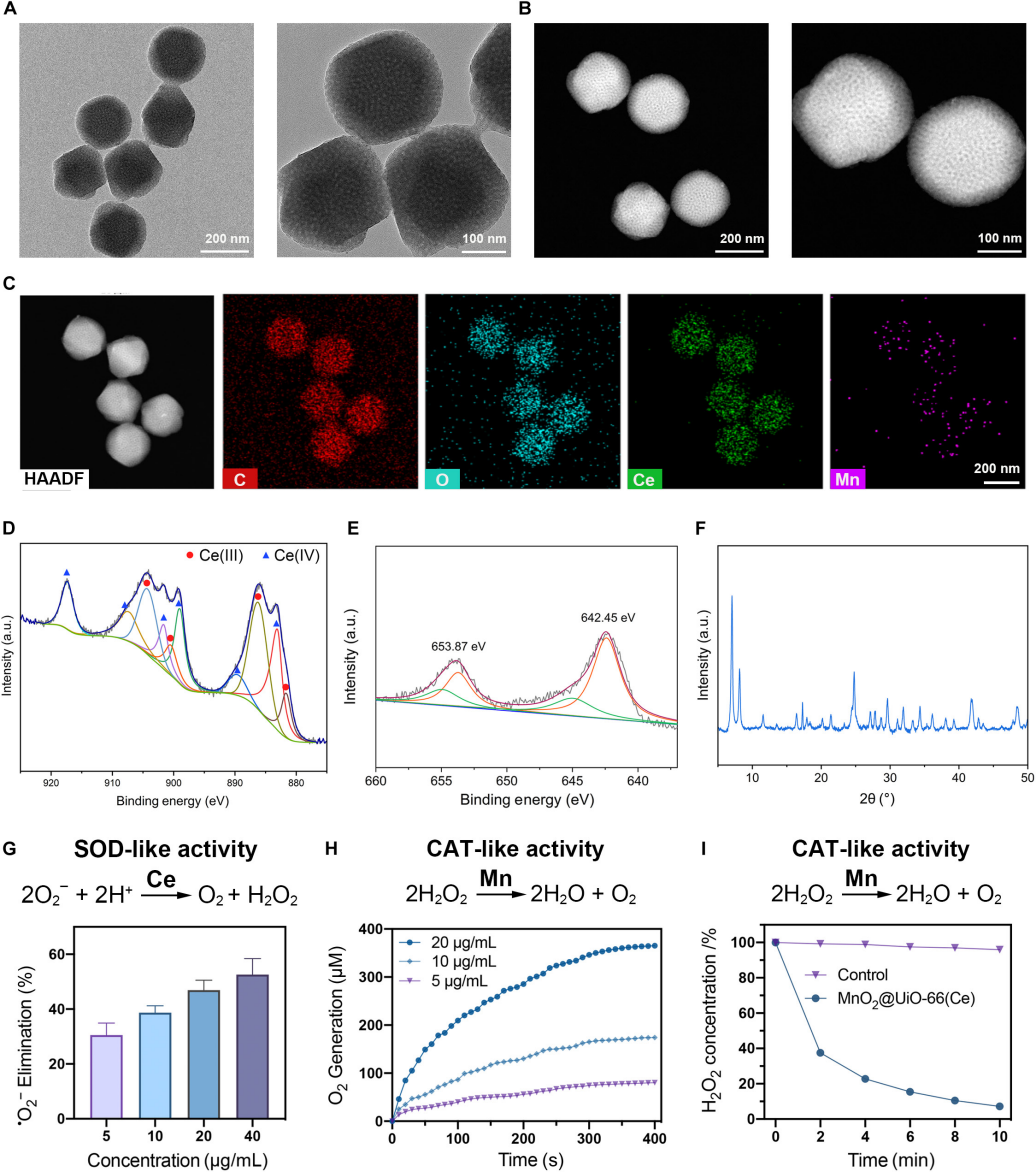
Characterization and enzyme-mimetic capacity of MnO_2_@UiO-66(Ce) (MCU). (A) Transmission electron microscopy (TEM) and (B) HAADF scanning TEM images of MCU. (C) Elemental distribution mapping analysis. (D) High-resolution x-ray photoelectron spectroscopy (XPS) spectra of the Ce3d regions. (E) Chemical state characterization of the Mn2p core levels by XPS. (F) X-ray diffraction pattern of MCU. (G) Scavenging capacity of •O^−2^ by MCU. SOD, superoxide dismutase; CAT, catalase. (H) Catalytic O_2_ production from H_2_O_2_ decomposition mediated by MCU. (I) Time-dependent H_2_O_2_ removal profile of MCU at 20 μg/mL concentration.

TEM and high-angle annular dark-field (HAADF) imaging confirmed the formation of well-defined octahedral MnO_2_@UiO-66 nanocrystals with homogeneous nanoparticle dispersion (Fig. 1A and B). These nanostructures displayed uniformly organized mesopores distributed throughout the particle architecture. TEM-based size analysis revealed an average particle diameter of approximately 230 nm, whereas hydrodynamic diameter measurements of around 275 nm were obtained via dynamic light scattering (Fig. S1). Quantitative elemental mapping confirmed the uniform spatial dispersion of C, O, Ce, and Mn inside the MCU architecture (Fig. 1C), confirming the uniform integration of MnO_2_ with cerium-based nodes within the mesoporous network.

XPS was utilized to characterize the chemical properties of MnO_2_@UiO-66. The Ce3d spectrum confirmed the coexistence of Ce^4+^/Ce^3+^ redox pairs, with characteristic Ce^4+^ signatures observed at binding energies of 917.3, 907.3, 901.6, 898.9, 889.6, and 883.1 eV. Corresponding Ce^3+^ contributions were identified at 904.2, 900.5, 886.2, and 881.6 eV (Fig. 1D). Analysis of the Mn2p region showed peaks at 642.45 eV (2p_3/2_) and 653.87 eV (2p_1/2_) (Fig. 1E), implying the successful reduction of KMnO_4_ to MnO_2_. Quantitative XPS assay determined surface atomic concentrations of 5.73% for Ce and 0.35% for Mn. The x-ray diffraction patterns confirmed that the crystalline framework of the MnO_2_@UiO-66 composite was preserved (Fig. 1F). Mesoporous nanostructures offer distinct advantages over exclusively microporous analogs, including facilitated mass transfer, increased exposure of catalytic centers, and enhanced catalytic performance [25].

The ROS elimination capacity of MCU was evaluated through multiple catalytic assays. Quantitative analysis of the SOD-like capacity was performed by assessing the elimination efficiency of superoxide anions (•O_2_^−^). The results revealed concentration-dependent •O_2_^−^ scavenging effects, and the maximum scavenging efficiency of nearly 40% was observed with 10 μg/mL MCU (Fig. 1G). For assessment of CAT-like activity, we measured O_2_ production from H_2_O_2_ decomposition. Although UiO-66 demonstrated rapid H_2_O_2_ adsorption capacity, it lacked catalytic conversion activity beyond the adsorption–desorption equilibrium within its mesochannels. The MCU composite synergistically combined the H_2_O_2_-capturing capability of UiO-66 with the CAT-mimetic ability of MnO_2_, resulting in accelerated O_2_ generation coupled with simultaneous H_2_O_2_ depletion upon treatment (Fig. 1H and I). These findings collectively establish MCU as a potent synergistic cascade system with complementary SOD- and CAT-mimetic properties.

### Biocompatibility of MCU

Since cytocompatibility is essential for the biological applications of nanomaterials, MCU cytocompatibility was assessed by CCK-8 experiments. The results showed that MCU did not significantly influence primary chondrocyte proliferation at the concentration of 1, 2, 4, and 8 μg/mL, whereas a mild toxic effect appeared at 16 μg/mL (Fig. S2A). Live and dead staining images presented no evident dead cells with MCU treatment ranging from 1 to 8 μg/mL (Fig. S2B). Therefore, the concentrations less than 8 μg/mL were determined safe and chosen for further biological applications.

### Cellular ROS-scavenging ability of MCU

**Fig. 2.**
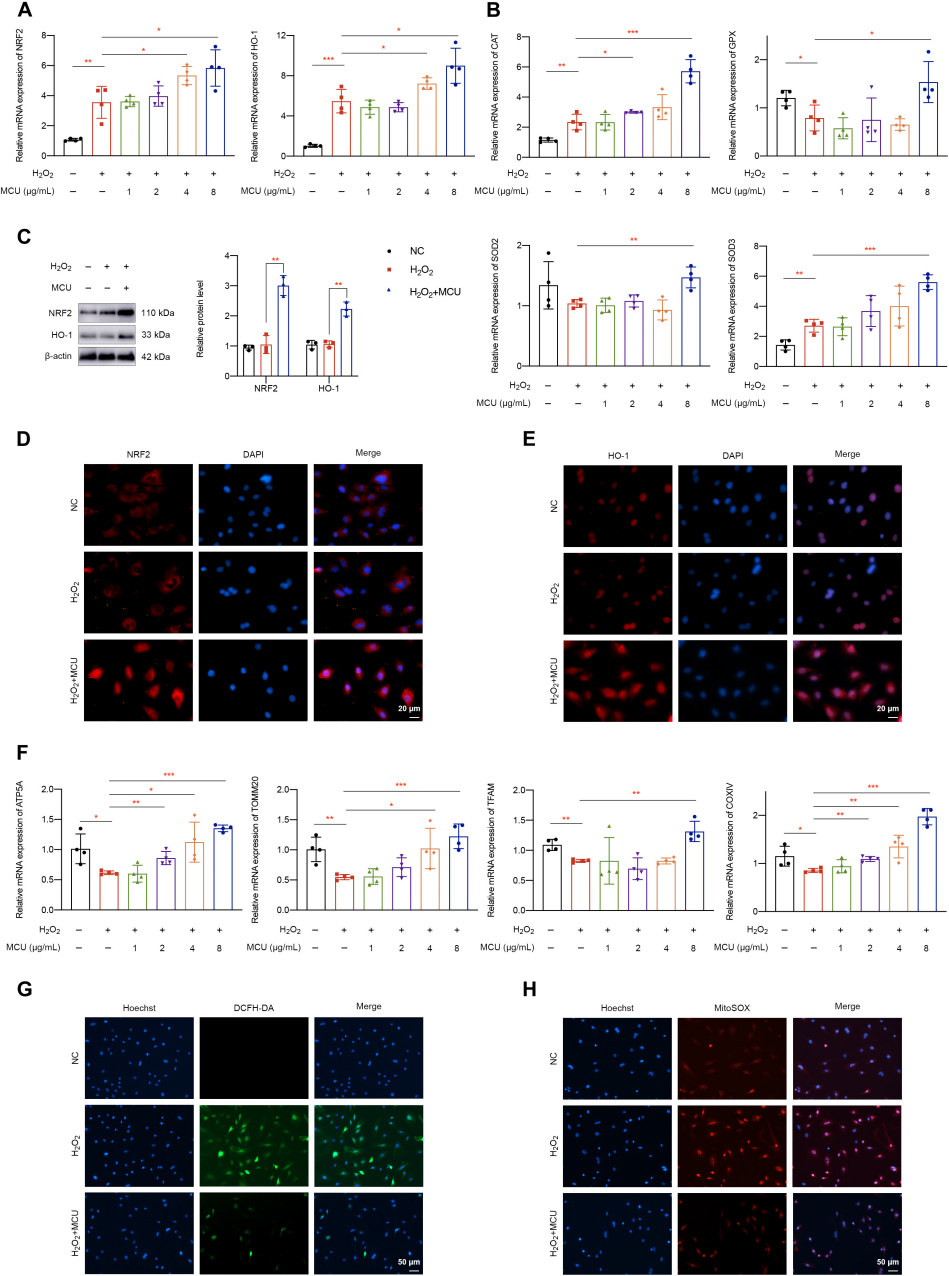
Reactive oxygen species (ROS)-scavenging ability of MCU. (A) qRT-PCR analysis of NRF2 and HO-1 in primary chondrocytes. *n* = 4. (B) qRT-PCR results of antioxidant-related genes in primary chondrocytes. *n* = 4. (C) Western blot assay of NRF2 and HO-1 protein levels in primary chondrocytes. *n* = 3. (D and E) Immunostaining images of NRF2 and HO-1. *n* = 3. (F) The mRNA levels of ATP5A, TOMM20, TFAM, and COXIV were regulated by MCU administration. *n* = 4. (G) DCFH-DA fluorescence assay to manifest intracellular ROS in chondrocytes upon different treatments. *n* = 3. (H) Representative MitoSOX staining images of chondrocytes after different treatments. *n* = 3. *P < 0.05, **P < 0.01, ***P < 0.001.

Recently, compelling evidence has uncovered the pathogenic role of redox imbalance in OA, where elevated oxidative stress triggers a catabolic/anabolic imbalance leading to extracellular matrix breakdown and chondrocyte dysfunction [26,27]. Consequently, therapeutic interventions targeting pathological ROS levels represent a promising strategy for OA management. The capacity of MCU to mitigate oxidative stress was evaluated through an in vitro cellular model of H_2_O_2_-induced oxidative damage. First, qRT-PCR and immunoblotting were used to quantify the mRNA and protein expression of oxidative stress-related regulatory genes, including NRF2, HO-1, CAT, GPX, SOD2, and SOD3. Quantitative analysis confirmed that MCU pretreatment elevated the expression levels of these markers, demonstrating that its functional enzyme-mimetic performance alleviated oxidative stress and the activation of an adaptive antioxidant response (Fig. 2A–C) [28]. In addition to its direct ROS-scavenging function, MCU exhibits a complementary mechanism that enhances intrinsic cellular defense programs, thereby supporting sustained tissue homeostasis. Additionally, consistent protein expression patterns were observed through immunofluorescence assay, which revealed markedly activated NRF2 and HO-1 signals in chondrocytes following MCU administration (Fig. 2D and E). Since mitochondria is the leading site of ROS production [29], representative markers of mitochondrial content were evaluated. The levels of ATP5A, TOMM20, TFAM, and COXIV decreased upon ROS induction, while MCU administration reversed the negative impact of oxidative stress on mitochondria (Fig. 2F). An intracellular ROS assay illustrated that pretreatment with MCU remarkably inhibits H_2_O_2_-induced intracellular ROS (Fig. 2G). Mitochondrial ROS (mtROS) play significant roles in the regulation of inflammatory processes, host defense mechanisms, and cellular activation, while their overproduction disrupts redox homeostasis, leading to impaired cellular function and the propagation of inflammatory tissue damage [30]. Meanwhile, the mtROS-scavenging capacity of MCU was assessed via MitoSOX Red staining, which revealed markedly suppressed mtROS generation in MCU-pretreated chondrocytes (Fig. 2H). Together, these data verify the efficacy of MCU in clearing pathological ROS and restoring redox homeostasis in primary chondrocytes.

### MCU prevents cartilage matrix degradation under an inflammatory microenvironment

**Fig. 3.**
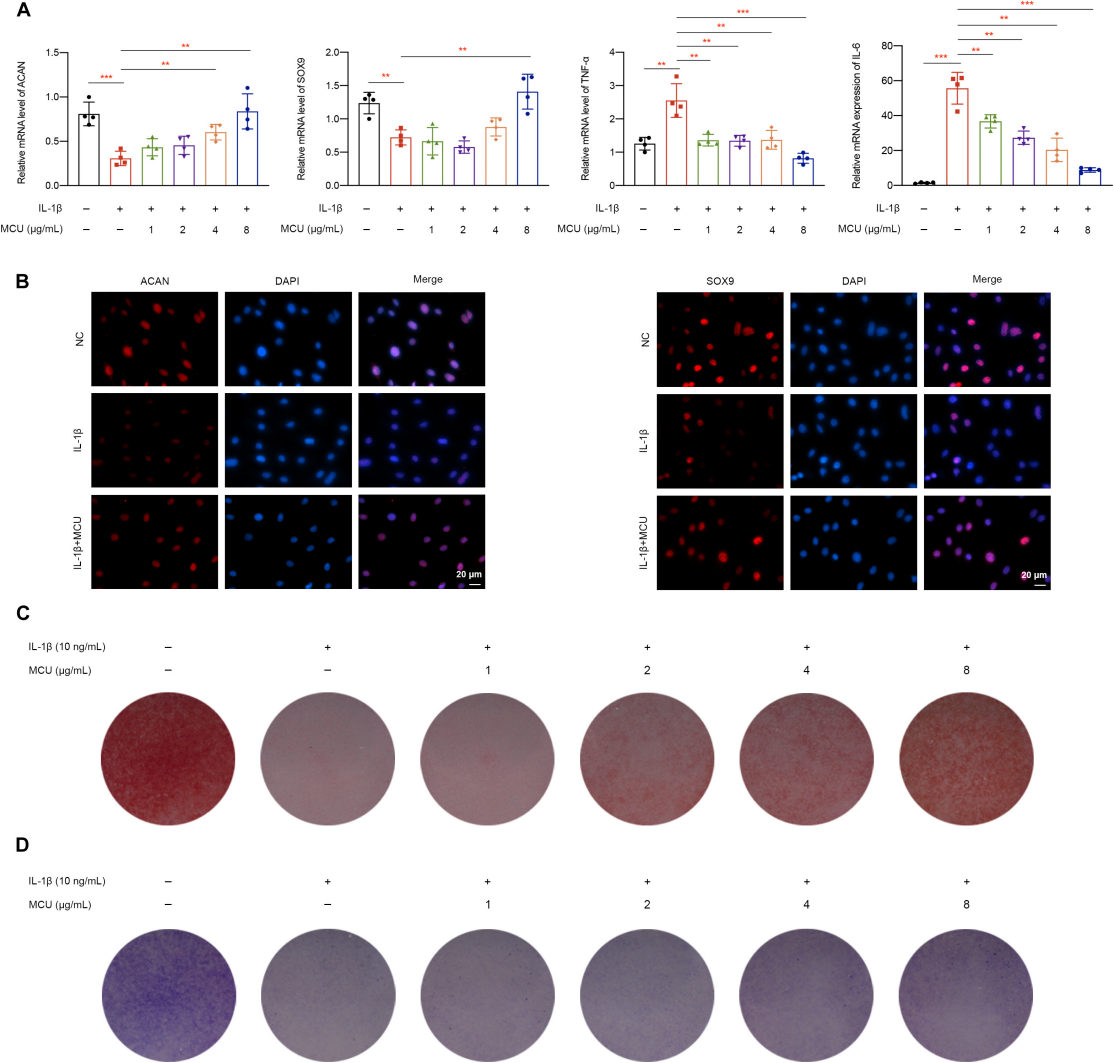
MCU prevents cartilage matrix degradation under inflammatory microenvironment. (A) qRT-PCR analysis results revealed that MCU effectively alleviates cartilage matrix breakdown and inflammatory reactions. *n* = 4. IL-1β, interleukin 1 beta; IL-6, interleukin 6. (B) Immunofluorescence staining intensity of ACAN and SOX9 was modulated by MCU. *n* = 3. (C and D) Safranin O staining and toluidine blue staining assay. *n* = 3. **P < 0.01, ***P < 0.001.

Oxidative stress acts as a principal pathogenic driver of OA pathogenesis [31]. To determine whether MCU could alleviate OA pathogenesis following oxidative stress mitigation, we detected representative cartilage-matrix-degradation markers and inflammatory factors. IL-1β stimulation markedly suppressed ACAN and SOX9 expression and induced tumor necrosis factor alpha and interleukin 6 expression, whereas MCU administration reversed this trend in a concentration-dependent manner; among the groups, the 8 μg/mL group presented the greatest effect (Fig. 3A). Similar expression patterns were illustrated by immunofluorescence staining of ACAN and SOX9 (Fig. 3B). Additionally, safranin O staining and toluidine blue staining revealed higher glycosaminoglycan (GAG) contents in MCU-treated chondrocytes compared to IL-1β stimulation group, with the 8 μg/mL MCU group exhibiting the highest GAG level (Fig. 3C and D). Consistent with our antioxidant activity data, these results revealed that 8 μg/mL MCU most effectively restores the physiological phenotype of primary chondrocytes under oxidative-stress conditions, thereby maintaining cartilage homeostasis. Future studies could focus on engineering stimuli-responsive materials for precise regulation of redox homeostasis. In addition, a detailed examination of MCU nanoparticle distribution and biological effects across various cell populations within the articular cavity microenvironment is needed to fully elucidate its comprehensive therapeutic mechanism.

### MCU activates AMPK signaling in OA chondrocytes

**Fig. 4.**
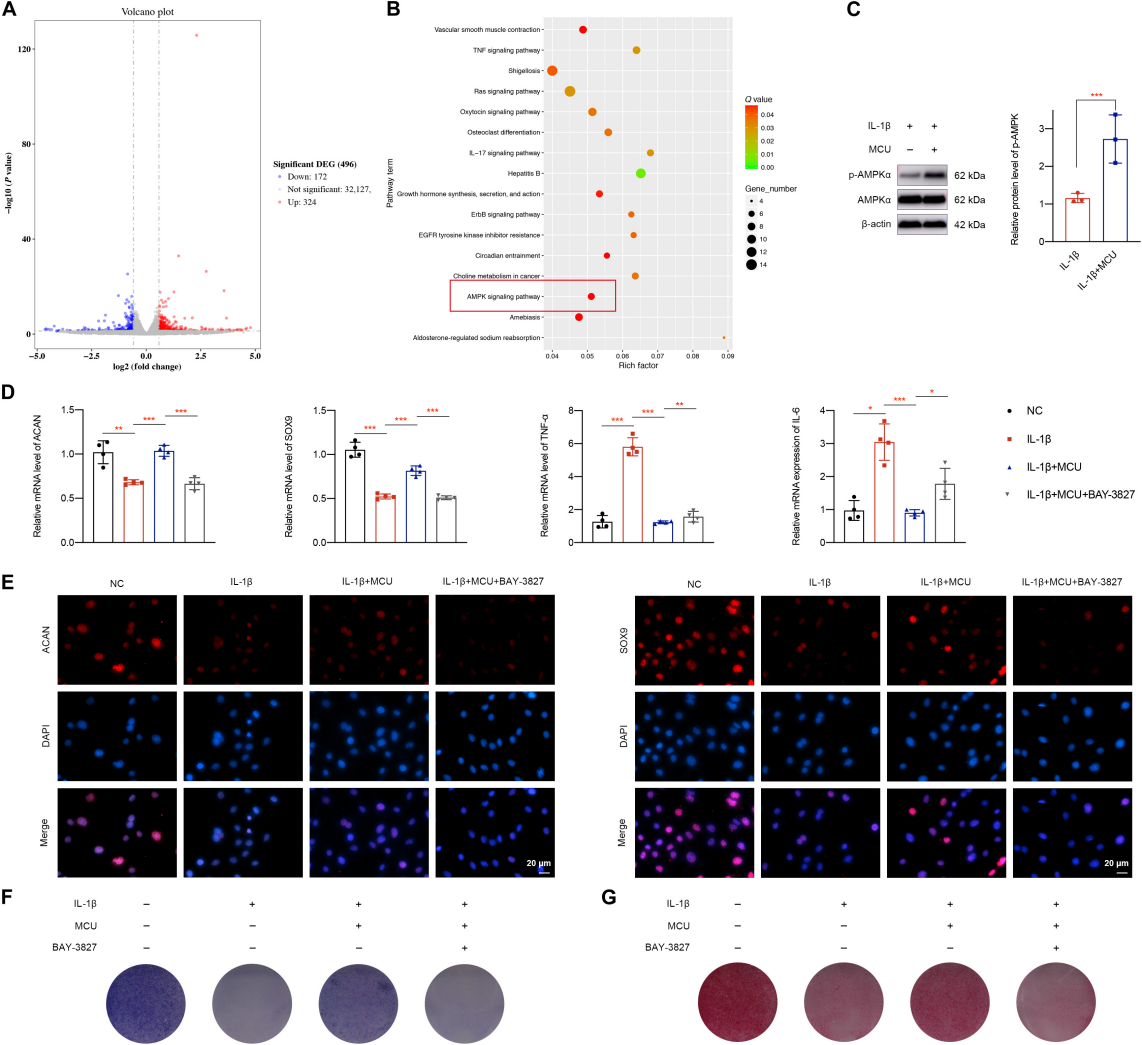
MCU stimulates adenosine monophosphate-activated protein kinase (AMPK) activation in osteoarthritis (OA) chondrocytes. (A) Volcano plot presenting differentially expressed genes (DEGs) regulated by MCU. (B) Kyoto Encyclopedia of Genes and Genomes pathway enrichment analysis. (C) Western blot and quantification of phosphorylated AMPK alpha (p-AMPKα) and AMPKα. *n* = 3. (D) qRT-PCR analysis of representative markers in primary chondrocytes during MCU treatment with AMPK signaling inhibition. *n* = 4. (E) Immunofluorescence imaging of ACAN and SOX9. *n* = 3. (F and G) Safranin O staining and toluidine blue staining results. *n* = 3. *P < 0.05, **P < 0.01, ***P < 0.001.

To elucidate the underlying mechanism of MCU in mitigating OA progression, RNA-seq was completed on IL-1β-stimulated primary chondrocytes with or without MCU administration. DEGs were identified on the basis of the threshold of *P* value <0.05 and |Fold change| > 1.5 (Fig. 4A). Kyoto Encyclopedia of Genes and Genomes pathway enrichment assay demonstrated an enrichment of DEGs in adenosine monophosphate-activated protein kinase (AMPK) signaling pathway (Fig. 4B). AMPK is a heterotrimeric serine/threonine protein kinase that serves as a central regulator of cellular energy homeostasis [32]. AMPK activation modulates a wide range of biological processes [33]. In addition to its canonical function in governing energy homeostasis, AMPK also has anti-inflammatory, antioxidant, and pro-regenerative effects [34,35]. Thus, we speculated that MCU may function through activating the AMPK signaling cascade. Western blot assay indicated an increase of phosphorylated AMPK upon MCU treatment (Fig. 4C). To further determine whether AMPK activation plays critical roles in MCU-mediated cartilage repair, the AMPK-specific inhibitor BAY-3827 was used. As illustrated in Fig. 4D to G, pretreatment with BAY-3827 markedly reduced the beneficial effects of MCU on cartilage matrix preservation and inflammation suppression, indicating that the protective effects of MCU on OA progression were diminished. These findings align with the reported role of AMPK signaling in OA cartilage that impaired AMPK activity can cause OA-like pathological changes [36]. Consistently, maintaining regular AMPK activity helps maintain cartilage matrix integrity under inflammatory conditions [37].

### MCU suppresses OA progression via the DOT1L–H3K79me3–β-catenin axis

**Fig. 5.**
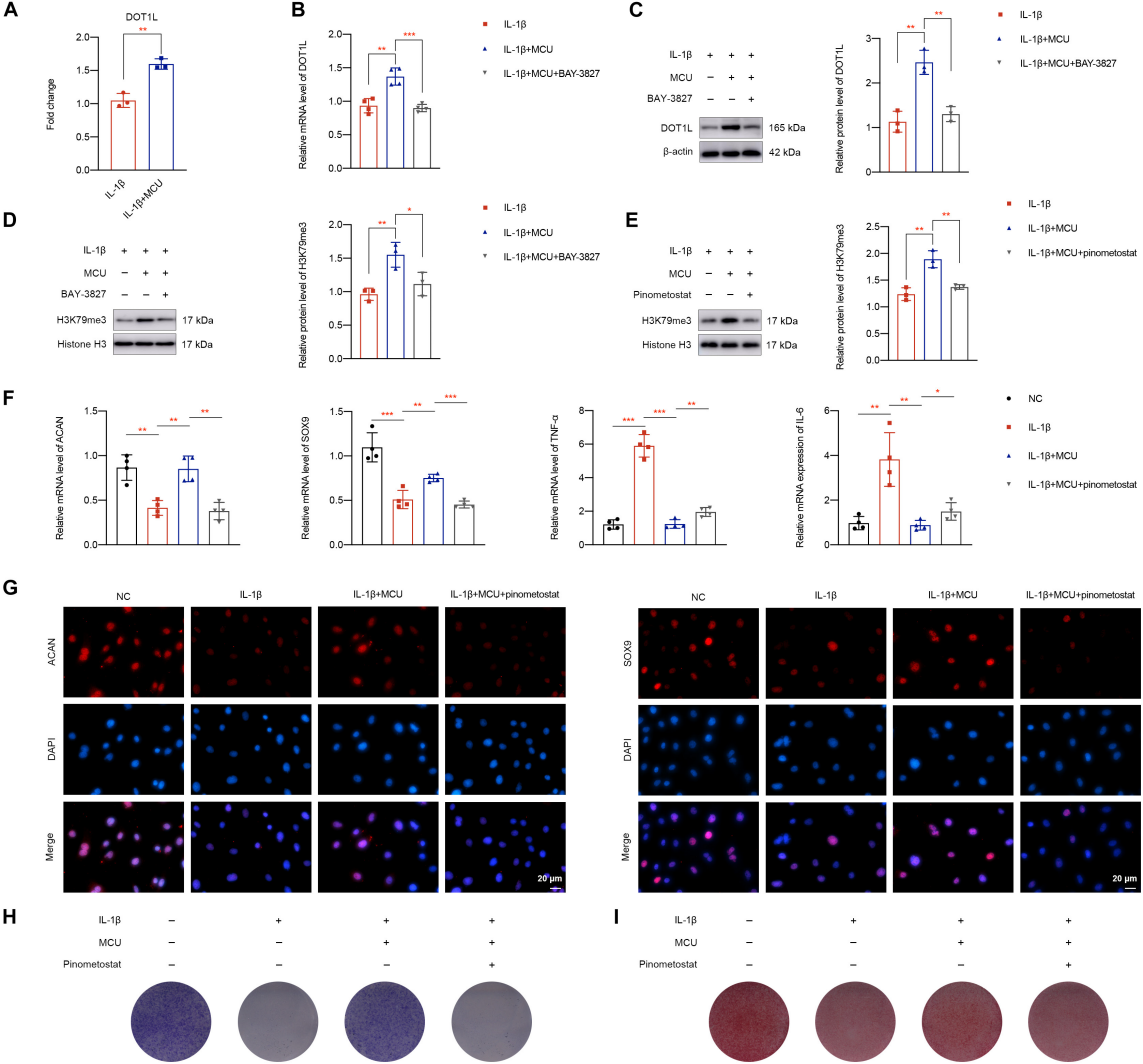
MCU mediates OA pathogenesis through disruptor of telomeric silencing 1-like (DOT1L)-dependent H3K79 methylation. (A) RNA-sequencing assay indicated a significant up-regulation of DOT1L by MCU treatment. *n* = 4. (B) The mRNA level of DOT1L was up-regulated in MCU-treated chondrocytes while AMPK inhibition reversed this effect. *n* = 4. (C) Western blot and quantification of DOT1L. *n* = 3. (D) AMPK inhibition reduced the protein level of H3K79me3. *n* = 3. (E) MCU modulated chondrocytes through DOT1L-mediated H3K79me3 activation. *n* = 3. (F) qRT-PCR analysis of representative markers. *n* = 4. (G) Immunofluorescence imaging of ACAN and SOX9. *n* = 3. (H and I) Safranin O staining and toluidine blue staining results. *n* = 3. *P < 0.05, **P < 0.01, ***P < 0.001.

Epigenetic modifications occur on genomic DNA and histones to modulate gene expression, which are closely related to human diseases [38]. Subsequently, we investigated whether epigenetic regulation involves in MCU-mediated OA therapy. Based on RNA-seq analysis results, we screened out disruptor of telomeric silencing 1-like (DOT1L), a class I-like *S*-adenosyl-l-methionine (SAM)-binding methyltransferase, which was significantly up-regulated upon MCU treatment (Fig. 5A) [39]. DOT1L is a conserved mammalian protein and the sole identified histone methyltransferase catalyzing H3K79 mono-, di-, and trimethylation, promoting target gene transcription [40]. DOT1L participates broadly in an array of fundamental biological processes, consequently involved in multiple disease progression [41]. Therefore, whether MCU regulates OA pathogenesis through DOT1L-mediated epigenetic regulation was investigated. qRT-PCR and western blot assay results verified the up-regulation of DOT1L expression in response to MCU treatment, whereas AMPK inhibition reversed this effect (Fig. 5B and C). These findings suggest that AMPK signaling is the upstream mediator of DOT1L activity. Meanwhile, the expression pattern of H3K79me3 was consistent with that of DOT1L, and DOT1L-specific inhibitor treatment could suppress H3K79me3 expression level, implying that DOT1L functions through the trimethylation of H3K79 (Fig. 5D and E). To further confirm the effect of DOT1L in OA, the specific DOT1L inhibitor, pinometostat, was employed. Pinometostat was reported to exhibit more than 37,000-fold selective for DOT1L over all other methyltransferases [42]. Importantly, pinometostat functions as a SAM competitive inhibitor that occupies the SAM binding pocket of DOT1L, a unique property among histone methyltransferases that further contributes to its target specificity [43]. The reduced cartilage matrix degradation and inflammatory responses in the MCU group were abolished by pinometostat pretreatment, indicating that MCU suppressed OA progression in a DOT1L-dependent manner (Fig. 5F–I). Collectively, our findings were consistent with those of previous studies, which indicated that DOT1L activity is essential for maintaining articular cartilage homeostasis [44,45]. It was reported that DOT1L deficiency promotes cartilage hypertrophy. Thus, maintaining or enhancing DOT1L activity may be an effective OA therapeutic strategy [46,47].

**Fig. 6.**
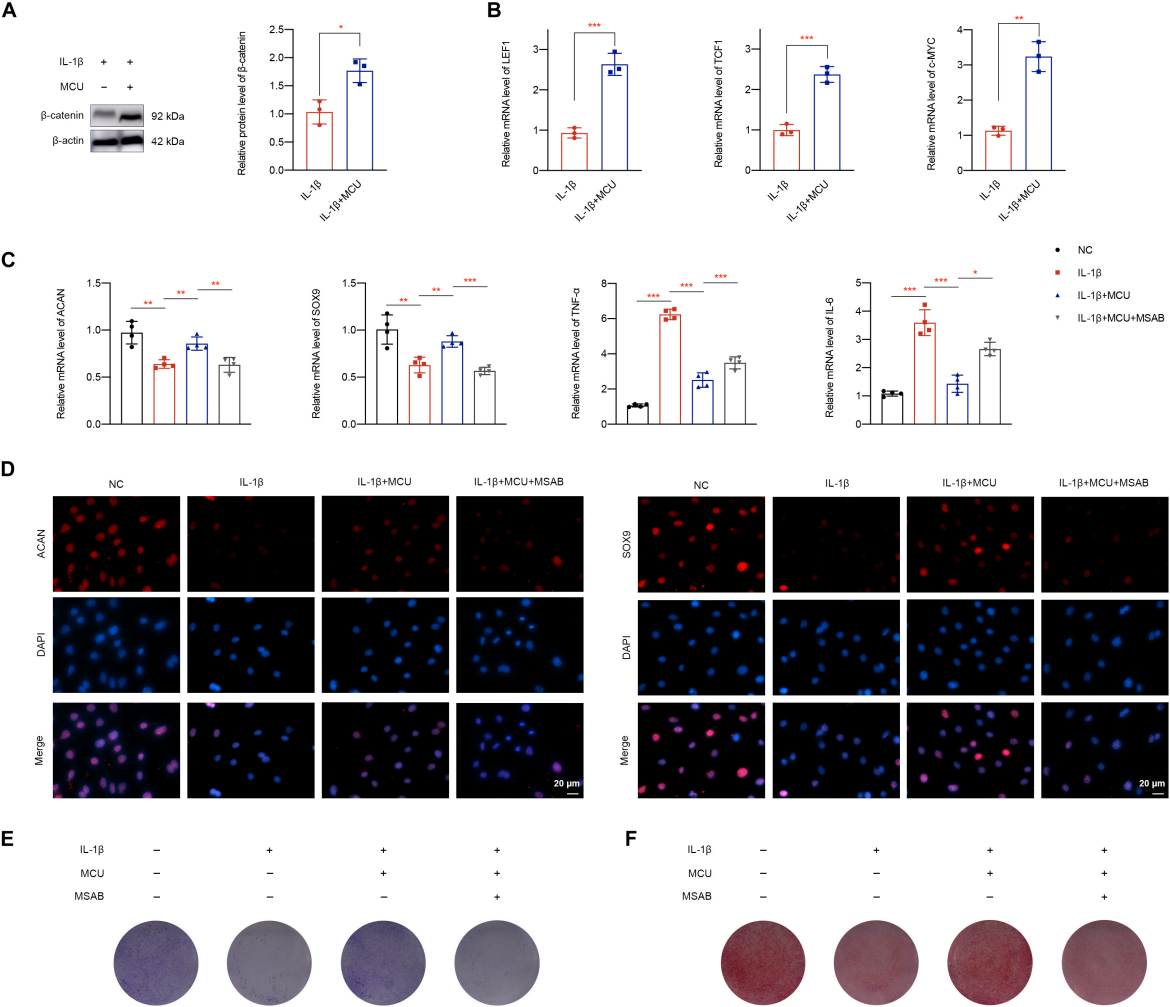
MCU alleviates OA progression via the epigenetic activation of the Wnt/β-catenin axis. (A) MCU activated β-catenin. *n* = 3. (B) Representative markers of Wnt/β-catenin downstream target genes. *n* = 4. (C) The mRNA expression of representative markers with the intervention of β-catenin inhibition under MCU administration. *n* = 4. (D) Immunofluorescence imaging of ACAN and SOX9. *n* = 3. (E and F) Safranin O staining and toluidine blue staining results. *n* = 3. *P < 0.05, **P < 0.01, ***P < 0.001.

To further elucidate the impact of DOT1L on the downstream target Wnt/β-catenin, we determined the levels of β-catenin and the pathway target genes (LEF1, TCF1, and c-MYC). The results revealed an increase of β-catenin and target gene expression upon MCU treatment (Fig. 6A and B). Concurrently, the reduced cartilage matrix degradation and inflammatory responses induced by MCU were attenuated by MSAB, a specific β-catenin inhibitor (Fig. 6C–F), underscoring the central role of this signaling axis in maintaining the therapeutic efficacy of MCU in the OA environment. A delicate equilibrium in Wnt pathway activity is critical for preserving joint tissue homeostasis [48]. Taken together, we speculated that MCU functions through activating AMPK signaling, which leads to β-catenin activation via the enhancement of the DOT1L–H3K79me3 axis. Our findings underscore the role of the AMPK–DOT1L–H3K79me3–β-catenin axis in the ability of MCU to reinstate cartilage homeostasis under oxidative stress.

### MCU alleviates OA pathogenesis in vivo

**Fig. 7.**
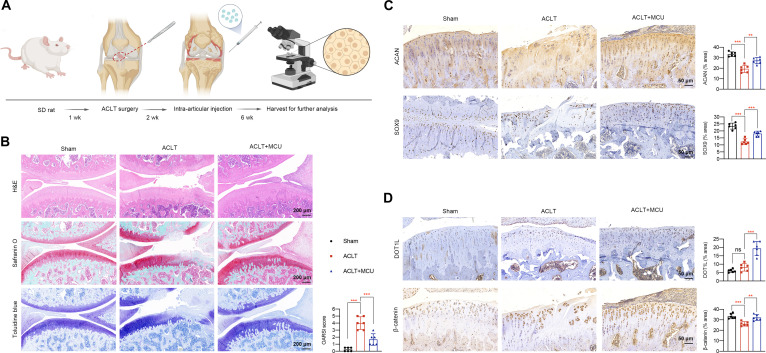
MCU effectively slows OA progression in vivo. (A) Schematic representation illustrating surgical OA induction in Sprague–Dawley (SD) rats via the anterior cruciate ligament transection (ACLT) method and the treatment protocol. (B) Hematoxylin and eosin (H&E), safranin O and fast green, and toluidine blue staining results of different samples and the corresponding Osteoarthritis Research Society International (OARSI) scores. *n* = 3. (C) Representative images of ACAN and SOX9 immunostaining and the relative staining intensities. *n* = 3. (D) Representative images of immunostaining of DOT1L and β-catenin in different samples and the relative staining intensities. *n* = 3. ns, not significant; **P < 0.01; ***P < 0.001.

**Fig. 8.**
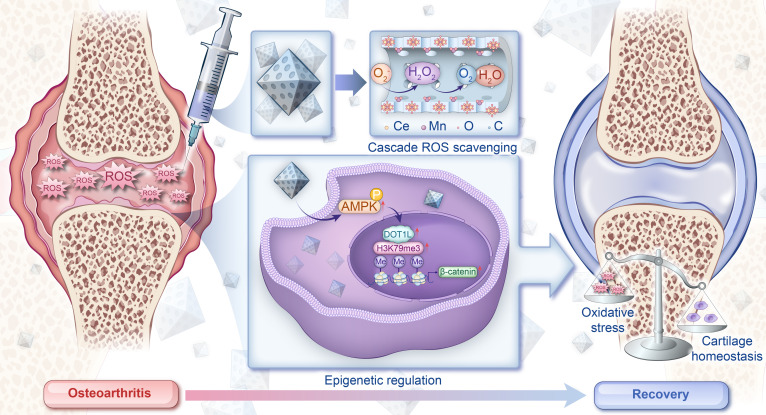
Schematic illustration of the underlying mechanism whereby MCU functions as an efficient ROS scavenger and epigenetic modulator for OA therapy.

The therapeutic efficacy of MCU was investigated in an experimentally induced OA model using SD rats subjected to ACLT surgery. At 2 weeks post-surgery, the rats in the MCU group were injected with MCU (8 μg/mL), and at 8 weeks post-surgery, the rats were euthanized for histological assessment (Fig. 7A). The comprehensive histological evaluation, including hematoxylin and eosin, safranin O–fast green, and toluidine blue staining, confirmed that MCU effectively inhibits OA progression (Fig. 7B). Specifically, a marked depletion of GAG and proteoglycan content was observed in cartilage tissues from the ACLT-induced model group, whereas MCU intervention partially restored these essential matrix components (Fig. 7B). Further analysis employing the Osteoarthritis Research Society International scoring system corroborated the efficacy of MCU therapeutic intervention in attenuating OA progression (Fig. 7B). Immunohistochemistry staining revealed elevated ACAN and SOX9 expression, confirming the in vivo biological effects of MCU (Fig. 7C). The above results indicated that MCU application promotes cartilage synthesis while suppressing cartilage catabolism. To verify the regulatory mechanism of MCU, immunohistochemistry of DOT1L and β-catenin was conducted. A significant increase in the expression of DOT1L and its downstream effector β-catenin was observed upon MCU administration compared with that in untreated group (Fig. 7D), supporting the hypothesis that MCU promotes transcriptional activation of the Wnt/β-catenin pathway through DOT1L-dependent H3K79 methylation. It is consistent with the established literature indicating that DOT1L protects against OA and could be an attractive therapeutic target [45,47]. Preservation or enhancement of DOT1L activity during aging or following joint injury may represent a viable strategy for OA prevention and treatment [46]. Overall, through its comprehensive ability to scavenge ROS, MCU has emerged as a promising therapeutic candidate for OA treatment. While our study firmly establish a functional link between MCU and the AMPK/DOT1L axis, we cannot exclude the possibility that other epigenetic regulators are also involved. The crosstalk between H3K79 methylation and other modifications may occur. Therefore, the potential contribution of other histone methyltransferases or demethylases in this process remains an important open question for future investigation. The proposed mechanistic framework underlying MCU-mediated OA therapy is summarized in Fig. 8. The features of MCU suggest its promise as a candidate for translation into clinical practice not only for OA but also for other ROS-related inflammatory diseases.

Although this study presents the development of a promising multifunctional nanozyme for effective OA therapy, certain limitations should be acknowledged. First, the current in vivo evaluation was confined to an 8-week period. Therefore, future studies should incorporate large-animal models with prolonged follow-up, allowing for a systemic evaluation of the degradation profile and long-term safety of MCU. Moreover, future investigations should address how nanozymes influence intercellular crosstalk among distinct articular cell populations, particularly the interplay between immune cells and chondrocytes. More importantly, pronounced interspecies differences in pharmacokinetic behavior exist, as rodents exhibit notably faster clearance rates than humans do [49]. Additionally, compared with human patients, mice exhibit distinct genomic and physiological responses to inflammatory stimuli and generally display greater resilience [50]. Therefore, a detailed pharmacokinetic analysis and a thorough assessment of the immune compatibility of MCU in higher-order animal species are imperative. Addressing these challenges is crucial for progressing MCU to human trials and ultimately achieving safe and effective OA therapy.

## Conclusion

In summary, this work presents a MnO_2_@UiO-66-based strategy for mitigation of the OA oxidative milieu, employing cascade reactions for broad-spectrum ROS clearance and cartilage matrix preservation, which collectively slow disease development. The MnO_2_@UiO-66 system is characterized by robust and complementary SOD-like and CAT-like enzymatic activities, enabling effective clearance of intracellular and mitochondrial ROS, consequently attenuating cartilage matrix destruction. In-depth mechanistic analysis indicated that MnO_2_@UiO-66 alleviates OA progression through AMPK–DOT1L-dependent H3K79 methylation, resulting in β-catenin signaling activation. Our findings provide a perspective for engineering multifunctional nanozymes capable of comprehensive ROS elimination, offering promising therapeutic strategies for other oxidative-stress-related inflammatory conditions.

## Data Availability

All data generated or analyzed during this study are included in this published article and its additional information files. All animal protocols of this study were approved by the Ethics Board of Shanghai Ninth People’s Hospital affiliated to Shanghai Jiao Tong University School of Medicine.
