# Supplementary material for: A Comprehensive Reactive-Oxygen-Species-Scavenging Metal–Organic Framework Orchestrates the AMPK–DOT1L–H3K79me3 Cascade to Alleviate Osteoarthritis
Source: Biomater Res. 2026 Aug 11;30:0402. doi: 10.34133/bmr.0402 (PMC13457904; doi:10.34133/bmr.0402)
Supplement: Supplementary file 1 [file bmr.0402.f1.docx]

**Supplementary materials**

**Table S1. The primers used for qRT-PCR analysis.**

| **Genes** | **Primer sequences** |
| --- | --- |
| Rat β-actin | forward 5’-GTAAAGACCTCTATGCCAACA-3’ |
|  | reverse 5’-GGACTCATCGTACTCCTGCT-3’ |
| Rat ACAN | forward 5’-TATGATGTCTACTGCTACGTGG-3’ |
|  | reverse 5’-GTAGAGGTAGACAGTTCTCACG-3’ |
| Rat SOX9 | forward 5’-GACAACTTTACCAGTTTCGGTC-3’ |
|  | reverse 5’-GAGGGAAAACAGAGAACGAAAC-3’ |
| Rat TNF-α | forward 5’-GCATGATCCGAGATGTGGAACTGG-3’ |
|  | reverse 5’-CGCCACGAGCAGGAATGAGAAG-3’ |
| Rat IL-6 | forward 5’-CCACTTCACAAGTCGGAGGCTTA-3’ |
|  | reverse 5’-GTGCATCATCGCTGTTCATACAATC-3’ |
| Rat NRF2 | forward 5’-TCTTGGAGTAAGTCGAGAAGTGT-3’ |
|  | reverse 5’-GTTGAAACTGAGCGAAAAAGGC-3’ |
| Rat HO-1 | forward 5’-AAGCCGAGAATGCTGAGTTCA-3’ |
|  | reverse 5’-GCCGTGTAGATATGGTACAAGGA-3’ |
| Rat GPX | forward 5’-CCTTTTAAGCAGTATGCAGGCA-3’ |
|  | reverse 5’-CAAGCCAAATGGCCCAAGTT-3’ |
| Rat CAT | forward 5’-AGCGACCAGATGAAGCAGTG-3’ |
|  | reverse 5’-TCCGCTCTCTGTCAAAGTGTG-3’ |
| Rat SOD2 | forward 5’-CAGACCTGCCTTACGACTATGG-3’ |
|  | reverse 5’-CTCGGTGGCGTTGAGATTGTT-3’ |
| Rat SOD3 | forward 5’-CCTTCTTGTTCTACGGCTTGC-3’ |
|  | reverse 5’-TCGCCTATCTTCTCAACCAGG-3’ |
| Rat ATP5A | forward 5’-TGCTTTCTTGTCTCATGTTGTG-3’ |
|  | reverse 5’-GTTTGTTACGATTTCCTTCAGC-3’ |
| Rat TOMM20 | forward 5’-CCAACTTCAAGAACAGGCTTCG-3’ |
|  | reverse 5’-CTGAGCACTGACAATTCTCTGAC-3’ |
| Rat TFAM | forward 5’-TTGGGCACAAGAAGCTGGTTAA-3’ |
|  | reverse 5’-GCATTCAGTGGGCAGAAGTCC-3’ |
| Rat COXIV | forward 5’-AAGAGCTACGTGTATGGCCC-3’ |
|  | reverse 5’-CCCTGAATGGGGTTGACCTT-3’ |
| Rat DOT1L | forward 5’-TGACCGCACCATACTTGAAAACTA-3’ |
|  | reverse 5’-GGCTCTTCTTGGACTTGCGAT-3’ |
| Rat LEF1 | forward 5’-TGTTTATCCCATCACGGGTGG-3’ |
|  | reverse 5’-CATGGAAGTGTCGCCTGACAG-3’ |
| Rat TCF1 | forward 5’-AGCTTTCTCCACTCTACGAACA-3’ |
|  | reverse 5’-AATCCAGAGAGATCGGGGGTC-3’ |
| Rat c-MYC | forward 5’-ATGCCCCTCAACGTGAACTTC-3’ |
|  | reverse 5’-CGCAACATAGGATGGAGAGCA-3’ |

**Supplementary Figures**

**Figure S1. Hydrodynamic particle size distribution by dynamic light scattering.**

**Figure S2. Cellular biocompatibility of MU.** (A) Cell proliferation of primary chondrocytes incubated with different concentrations of MCU after 24, 48 and 72 h by CCK-8 assay.

(B) Representative Live&Dead staining images of primary chondrocytes treated with

different concentrations of MCU. ***p < 0.001.
